# Modeling of Large Pharmacokinetic Data Using Nonlinear Mixed‐Effects: A Paradigm Shift in Veterinary Pharmacology. A Case Study With Robenacoxib in Cats

**DOI:** 10.1002/psp4.12141

**Published:** 2016-10-22

**Authors:** L Pelligand, A Soubret, JN King, J Elliott, JP Mochel

**Affiliations:** ^1^ Royal Veterinary CollegeHatfieldUnited Kingdom; ^2^ Department of PharmacometricsNovartis PharmaceuticalsBaselSwitzerland; ^3^ Elanco Animal Health Inc.BaselSwitzerland

## Abstract

The objective of this study was to model the pharmacokinetics (PKs) of robenacoxib in cats using a nonlinear mixed‐effects (NLME) approach, leveraging all available information collected from cats receiving robenacoxib s.c. and/or i.v.: 47 densely sampled laboratory cats and 36 clinical cats sparsely sampled preoperatively. Data from both routes were modeled sequentially using Monolix 4.3.2. Influence of parameter correlations and available covariates (age, gender, bodyweight, and anesthesia) on population parameter estimates were evaluated by using multiple samples from the posterior distribution of the random effects. A bicompartmental disposition model with simultaneous zero and first‐order absorption best described robenacoxib PKs in blood. Clearance was 0.502 L/kg/h and the bioavailability was high (78%). The absorption constant point estimate (K_a_ = 0.68 h^−1^) was lower than beta (median, 1.08 h^−1^), unveiling flip‐flop kinetics. No dosing adjustment based on available covariates information is advocated. This modeling work constitutes the first application of NLME in a large feline population.

**Study Highlights**

**WHAT IS THE CURRENT KNOWLEDGE ON THE TOPIC?**

☑ Robenacoxib is a cyclooxygenase‐2 selective NSAID registered for use in cats. In veterinary medicine, characterization of the PKs is usually performed using a two‐stage approach limiting most of the analyses to rich datasets.

**WHAT QUESTION DID THIS STUDY ADDRESS?**

☑ The objective of this study was to model the PKs of robenacoxib using NLME to leverage the variety of information obtained from sparse and rich datasets for the appropriate assessment of the drug kinetics and its between‐subject variability in cats.

**WHAT THIS STUDY ADDS TO OUR KNOWLEDGE**

☑ This work constitutes the first population PK analysis at a large scale in cats. Simultaneous fitting of all dosing routes unveiled the flip‐flop kinetics of robenacoxib for which no dosing adjustment seems necessary. Using several samples of the posterior distribution instead of the EBE allows for a better estimation of the correlation between model parameters.

☑ **HOW MIGHT THIS CHANGE DRUG DISCOVERY, DEVELOPMENT, AND/OR THERAPEUTICS?**

This research illustrates the value of NLME for the reconciliation of diverse PK data in veterinary drug research and development.

Despite a few available examples in the literature,^1^, ^2^, ^3^ the use of nonlinear mixed effect (NLME) for the modeling of pharmacokinetic (PK) data remains marginal in veterinary medicine. Noncompartmental or compartmental analysis of single studies, including, on average, 8–12 animals, are still standard practices in the veterinary literature or dossier submission from industry for market approval. Therefore, NLME models are not currently used to their full potential in veterinary pharmacology.

Sampling from a clinical population is essential to verify the PK model validity established from preclinical experiments, especially if this was obtained from healthy, and usually young, laboratory animals, which are likely not to be representative of the target population. Optimization for best use of PK data obtained from veterinary patients involved in clinical trials is of critical importance because the density of data obtainable is essentially sparse for two main reasons. First, the patients involved in clinical studies are client‐owned, thus, the number of venipunctures allowable is strictly scrutinized and capped for ethical and welfare reasons. Second, in animals of small size, such as the cat, the volume of blood that can be sampled is limited. Considering these two limitations, sampling small veterinary patients, such as cats, presents comparable challenges to the ones encountered in clinical PK studies in neonates.

We propose a novel application of population PK to a combination of sparse and rich data collected in the context of veterinary practice in cats using the example of robenacoxib (a cyclooxygenase‐2 highly selective nonsteroidal anti‐inflammatory drug (NSAID) licensed for use in cats and dogs). Our objective was to evaluate whether clinical cats undergoing ovariohysterectomy surgery had the same robenacoxib exposure in the perioperative period than healthy laboratory cats. General anesthesia can, through changes in cardiac output and fluid balance, alter the clearance or distribution, therefore, changing the blood or plasma concentration time curve and the duration of effect. There is currently very little relevant evidence in veterinary species documenting the effect of perioperative care, general anesthesia, and timing of drug administration with regard to the PK of an analgesic drug.^4^, ^5^ Our hypothesis was that there would be no difference in exposure of robenacoxib in the perioperative period.

In the present paper, we report the population PK model and its covariate analysis for robenacoxib using a pooled dataset from 83 cats (97 administrations, either s.c. or i.v. routes) either densely (laboratory cats) or sparsely sampled (veterinary patients undergoing ovariohysterectomy for neutering). Our analysis further illustrates how screening for parameter correlations can be made more robust by using multiple samples instead of just the mode (i.e., the empirical Bayes estimate (EBE)) of the posterior distribution. In essence, this paper outlines how sparse data obtained in veterinary clinical studies can be leveraged to better understand drug PKs in actual clinical practice.

## METHODS

Blood robenacoxib concentrations were obtained from eight different studies in which cats were administered robenacoxib i.v. or s.c. during the drug development or the postmarketing phases. Blood concentration time profiles were available from 83 cats. The demographics of the cats, dose/route of administration, and design of these studies (rich or sparse sampling strategy) are summarized in **Table** 1.^6^, ^7^, ^8^, ^9^, ^10^, ^11^ Fourteen of these 83 cats received robenacoxib by both routes. Robenacoxib was administered i.v. to 23 cats and s.c. to 74 cats. Forty‐seven cats were densely sampled (between 9 and 12 blood samples after administration). Thirty‐six cats included in a clinical study were sparsely sampled (between 1 and 2 samples per cat).

**Table 1:** List of PK studies including cats that were dosed intravenously (IV) or subcutaneously (SC), with reference from the literature.

Study	Population	Weight range (kg)	Age range (y.o.)	Number of periods (route)	Dose (mg/kg)	Sampling strategy	Sampling schedule	Reference
CRA 03/182	5m/5f = 10 cats	3.1–4.1	1.2–2.2	1 (SC)	2	Dense	0, 5, 15, 30, 45 min, 1, 2, 4, 6, 8, 12, and 23h	(Giraudel et al. 8 2009)
CRA 04/034	2m/1f = 3 cats	3.2–5.7	4.8–6.2	1 (IV)	1.94–1.97	Dense	0, 5, 30 min, 1, 2, 4, 6, 8, 24h	Unpublished
CRA 04/094	6m/6f = 12 cats	2.3–5.1	0.96–1.01	2 (IV/SC)	1.64–2	Dense	0, 3, 15, 30 min, 1, 2, 3, 4, 5, 6, 8h	(King *et al*. 9 2013)
CRA 07/137	3m/3f = 6 cats	3.1–4.5	2.5–3.3	1 (IV)	2	Dense	0, 5, 15, 30min, 1, 1.5, 2, 3, 4, 6, 9h	(Pelligand *et al*. 7 2012)
CRA 08/124	3m/3f = 6 cats	3.7–4.8	1.26–1.45	1 (SC)	2	Dense	0, 15, 30 min, 1, 2, 3, 4, 6, 9, 12, 24h	(Pelligand *et al*. 7 2012)
CRA 08/189	1m/1f = 2 cats	3.4–3.8	1.55	2 (IV/SC)	2.1	Dense	0, 5, 15, 30 min, 1, 2, 3, 4, 6, 9, 12h	(Pelligand *et al*. 7 2012)
CRA 09/209	5m/3f = 8 cats	3.4–4.5	1.2–4.3	1 (SC)	2 to 2.2	Dense	0, 5, 15, 30 min, 1, 1.5, 2, 3, 4, 6, 9, 12h	(Pelligand *et al*. 6 2014)
Perioperative study	36f = 36 cats	1.8–4	0.34–4.3	1 (SC)	2	Sparse	Postoperatively: one sample at extubation (recovery) and one sample 2h thereafter	(Pelligand *et al*. 10 2015a; Pelligand *et al*. 11 2015b)

m=male, f=female

### Animal phases for rich sampling dataset

The studies whose names started with CRA were carried out in healthy, conscious, domestic shorthair cats and published elsewhere (**Table** 1). The cats weighed between 2.3 and 5.7 kg. The cats were either loose‐housed in a research colony (between experiments) or kept in individual stainless steel cages (during experimental phases). The cats were fed a commercial dry food diet once or twice a day with the second meal of the day given after the last sample of the day. Cats were weighed on the day of administration to calculate the exact dose of robenacoxib to be administered by the i.v. and s.c. (between scapula) routes. Venous blood samples were collected from preplaced jugular catheters or by repeated venipuncture. The washout period between robenacoxib administrations was at least 1 week.

### Animal phase for sparse sampling dataset

Samples were obtained from a population of 36 clinical female cats admitted for elective ovariohysterectomy. After intramuscular premedication with buprenorphine and acepromazine, anesthesia was induced with propofol and maintained with isoflurane following endotracheal intubation. The cats were administered 2 mg/kg robenacoxib s.c., at various times before the surgical procedure or at the end of the surgical procedure (extubation). For the 24 cats that received robenacoxib preoperatively, two blood samples were taken (on extubation and 2 hours after). In the 12 cats that received robenacoxib at the end of the surgical procedure, only one blood sample was taken (2 hours after extubation).

### Ethical and regulatory approvals

All studies were approved by the local animal ethics and welfare committees and complied with national regulations of France, Switzerland, or the United Kingdom. All studies complied with the Guidelines for the Conduct of Pharmacokinetic Studies in Target Animal Species (EMEA/CVMP/139/99‐Final) and the guideline for the Conduct of Efficacy Studies for Non‐Steroidal Anti‐Inflammatory Drugs (EMEA/CVMP/237/01).

### Analytical phase

Blood samples (∼1.5 mL) were collected in EDTA tubes and stored between −20°C to −80°C until assayed. Robenacoxib concentrations in feline blood were measured using a sensitive analytical method, as described by Jung *et al*.^12^ Briefly, the method involved an initial analysis by high‐performance liquid chromatography‐UV, covering the range of 500–20,000 ng/mL and, if required, a subsequent analysis by liquid chromatography mass spectrometry, covering the range of 3–100 ng/mL (lower and upper limits of quantification) for blood. Depending on the results obtained by UV analysis, samples were diluted if necessary in order not to exceed a concentration of 100 ng/mL in the mass spectrometry method. Handling of the below limit of quantification values (55 of 652 measured concentrations) is described thereafter. All analyses complied with the guideline on bioanalytical method validation.^13^

### Data analysis and model evaluation

Population PK modeling from isolated or combined routes of administration was performed using the stochastic approximation expectation maximization (SAEM) algorithm for nonlinear mixed‐effects models implemented in Monolix version 4.3.2 (Lixoft, France).

Sequential fitting was used to test a series of rival models of absorption. The fixed‐effects parameters of the disposition function (clearance (CL), volume of the central compartment (V1), intercompartmental clearance (Q), and volume of the central compartment (V2)) estimated from the separate fitting of the i.v. dosing were fixed for the following fitting of the s.c. and i.v. data. The random effects were estimated from the fitting of the full set of information.

The convergence of the SAEM algorithm was assessed by inspection of the stability of the fixed‐ and random‐effect parameters and the log‐likelihood estimate after the exploratory period of the algorithm (i.e., after 1,000 iterations of SAEM). Then, the standard goodness‐of‐fit diagnostics, including population and individual predictions vs. observations, and the distributions of weighted residuals and normalized prediction distribution errors over time were used to evaluate the performances of the candidate models. Residual error estimates from the mathematical models were used as supportive information for evaluation of lack of fit. Normality and independence of residuals were evaluated using histograms, quantile‐quantile plots, and autocorrelation of conditional weighted residuals.

For converging models with satisfactory goodness‐of‐fit diagnostics, model selection was based on the Bayesian information criteria (BIC) and the precision of the model parameter estimates (i.e., in particular during the covariate search, in which models where the parameter precision estimates could not be assessed were discarded from the analysis). The BIC was selected over the Akaike Information Criterion as it tends to select simpler and more parsimonious models.

### PK model development

Similar to Sheiner and Ludden,^14^ mathematical models were written using the following format as the basic model:

$$y_{ij}=F\left(\phi_{i},t_{ij}\right)\times \left(1+b\;\varepsilon_{ij}^{b}\right)+a\;\varepsilon_{ij}^{a},\;j=1,\ldots ,n_{i}$$

ϕi=FikaiCLiV1iQiV2i=invlogit(logitμF+ηF,i)μkai·eηkaiμCL·eηCL,iμV1·eηV1,iμQ·eηQ,iμV2·eηV2,i, i=1,…,N

where $y_{ij}$ is the observed variable (i.e., robenacoxib blood concentration) measured on the ith individual at time $t_{ij}$, $\phi_{i}$ is the vector of individual parameters, $F(\phi_{i},t_{ij})$ is the value of that observed variable at time $t_{ij}$ for an individual with parameters $\phi_{i}$, and $\varepsilon_{ij}^{a}$ and $\varepsilon_{ij}^{b}$ are independent random variable normally distributed around zero with a variance of one.

In NLMEs, $F\left(\phi_{i},t_{ij}\right)$ is known as the structural model (error‐free), whereas the terms $a\;\varepsilon_{ij}^{a}$ and $b\;\varepsilon_{ij}^{b}\;F\left(\phi_{i},t_{ij}\right)$ are describing an additive and proportional residual error model (combining unexplained variability and measurement noise) parametrized by the SD a and b. The μ represents the typical value (population median) of a model parameter. The sources of variation between the individual parameters $\phi_{i}$ can be further explained by population characteristics (i.e., covariates) that can be included additively or proportionally to μ. The independent random variables ηi represent the unexplained difference between the value of the individual parameters $\phi_{i}$ and their median μ. The random variables $\;\eta_{i}$ are assumed to be normally distributed with mean value 0 and variance‐covariance matrix Ω (implying that $\phi_{i}$ are log‐normally distributed). For the bioavailability model parameter, a logit transform function was used to insure that the estimated value was bounded between zero and one.

### Inclusion of data below the limit of quantification

Monolix handles the values below limit of quantification by adding to the likelihood a term describing the probability that the true observation lies between zero and the limit of quantification. For the calculation of the likelihood, this is equivalent to the M3 method implemented in NONMEM and described by Beal^15^ (2001).

### Parameter correlation estimates

Correlations between model parameters were first explored through visual inspection of the eta vs. eta scatterplot. Then, similar to Lavielle and Ribba,^16^ several samples of the posterior distribution obtained at the last iteration of the SAEM algorithm, rather than the EBE, were used to assess the correlation between model parameters. Therefore, because 10 different Markov chains were used during the estimation step, 10 samples of the posterior distribution were obtained for each individual.

### Inclusion of covariate relationships

The effect of two continuous variables (weight (range, 1.77–5.7 kg), age (range, 0.34–6.1 years)) and two factors (gender and perioperative status) on CL, V1, and bioavailability (Ftot) were evaluated herein. Age and bodyweight were normalized by their median value and log‐transformed during the covariate analysis (thereafter referred to as t_AGE and t_WEIGHT0, respectively).

To confirm the significant impact of the covariates selected by the graphical analysis, a backward elimination procedure was performed to compare the different models using the BIC=‐2*LL+log(n)P criteria where LL is the log‐likelihood estimate, P is the total number of parameters to be estimated, and n is the number of subjects. Using the BIC criteria during the backward elimination is equivalent to selecting a sample size‐dependent threshold criteria (i.e., as it behaves like a significance test with the first type error alpha (α) depending on the sample size; e.g., α = 0.13 for *n* = 10, α = 0.032 for *n* = 100, and α = 0.0086 for *n* = 1,000).^17^ The full set of covariates (ANEST, t_AGE, t_WEIGHT0, and GENDER) was tested on the following model parameters: CL, V1, and F. The selection of the relevant covariates was based on the results from the backward elimination step using Monolix for each model fit.

### Model validation

To assess the validity of final model parameter estimates, the 95% confidence intervals of the 10th, 50th, and 90th percentiles calculated from 500 Monte‐Carlo simulations were overlaid to the corresponding percentiles of the raw data using the visual predictive check option in Monolix version 4.3.2.

## RESULTS

### Study demographics

Data from 83 cats were pooled together from eight robenacoxib PK studies (**Table** 1). The dose ranged from 1.6–2.3 mg/kg. Among the laboratory cats, 22 were females and 27 were males. The median bodyweight was 3.9 kg (interquartile range, 3.45–4.14). The weight extremes were 2.5 and 5.7 kg. The median age was 1.32 years old (interquartile range, 1.0–1.55), with age extremes of 0.97–6.1 years. The 36 female cats from the clinical study were sparsely sampled: the time of blood sample collection spanned between 0.8 and 8.2 hours after robenacoxib administration. The median bodyweight of these cats was 2.73 kg (interquartile range, 2.41–2.99). The weight extremes were 1.77 and 4.0 kg. The median age was 0.76 years (39 weeks; interquartile range, 0.46–1.195). The age extremes were 0.34 (17.7 weeks) and 4.31 years old.

### PKs

**Figure 1:**
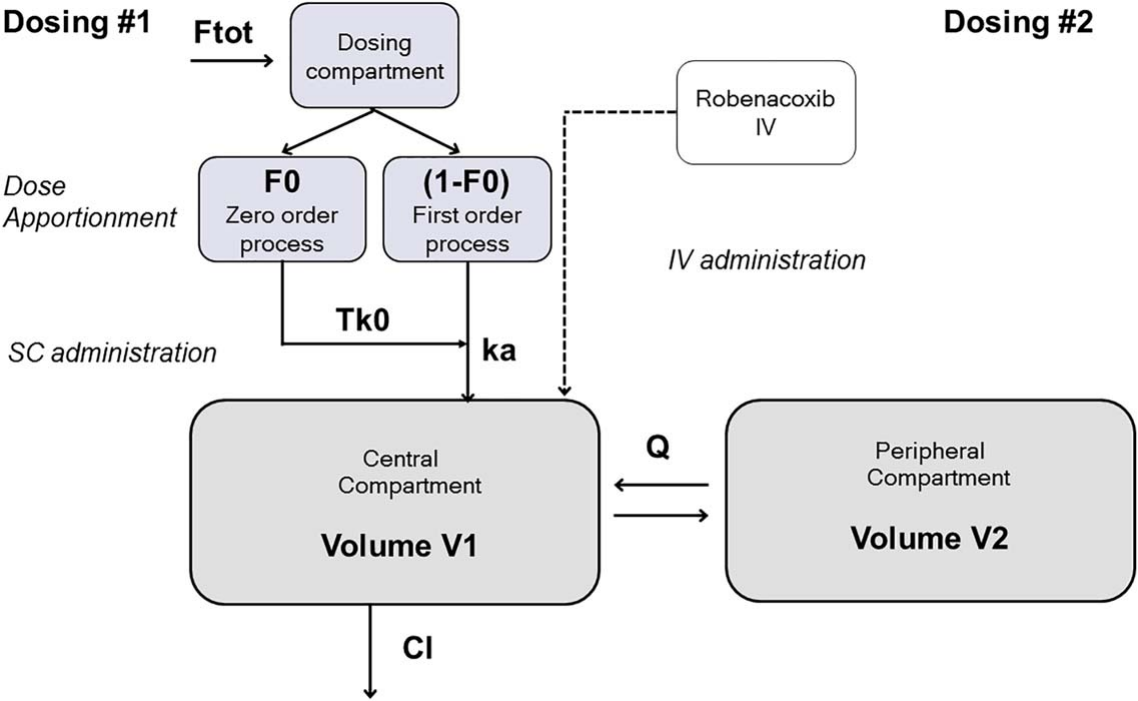
Schematic of robenacoxib final pharmacokinetic model following i.v. and s.c. dosing in cats. The fixed‐effects parameters of the disposition function (CL, V1, Q, V2) estimated from the separate fitting of the intravenous dosing were fixed for the following fitting of the subcutaneous and intravenous data. The random effects were estimated from the fitting of the full set of information. More details on the abbreviated parameters can be found in Table 2. Ftot, bioavailability; ka, absorption rate; Q, intercompartmental clearance; Tko, absorption duration (0‐order).

A two‐compartment mammillary disposition model with first‐order elimination and simultaneous first‐order and zero‐order (Tk0) absorption best described the PK of robenacoxib in blood (**Figure** 1, Supplementary Figure S3). Adding a correlation between ka and Tk0 (corr = −0.24) yielded the best objective function and BIC value and was, therefore, included in the model structure.

**Figure 2:**
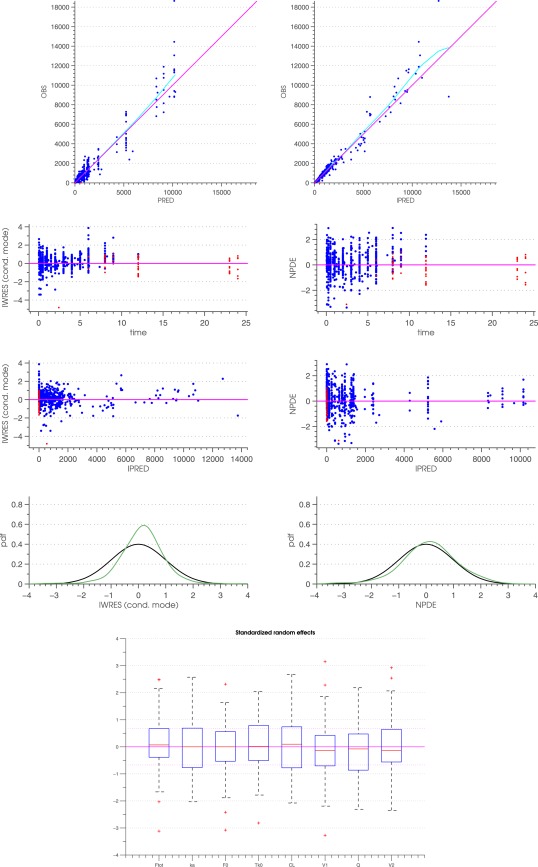
Model evaluation: Standard Goodness‐Of‐Fit diagnostics and Standardized Random Effects. Top pane: Population predictions vs. observations (left) and individual predictions vs. observations (right). The purple line represents the identity line; the regression line is portrayed in light blue color; points below quantification limits are represented with red dots. Middle pane first row: IWRES vs. time (left) and NPDE vs. time (right); Middle plane second row: IWRES vs. IPRED (left) and NPDE vs. IPRED (right); Middle pane third row: Theoretical and computed pdf vs IWRES (left) and pdf vs. NPDE (right). Points below the quantification limits are represented with red dots. Bottom pane: Box plots of the standardised random effect distribution. Interquartile range (IQR) together with the median. Dotted lines represent ± 1.5 x IQR from the first and the third quartile. Outliers are represented with red crosses. IWRES, individual weighted residuals; NPDE, normalized prediction distribution error; OBS, observed values; pdf, probability density function.

**Figure 3:**
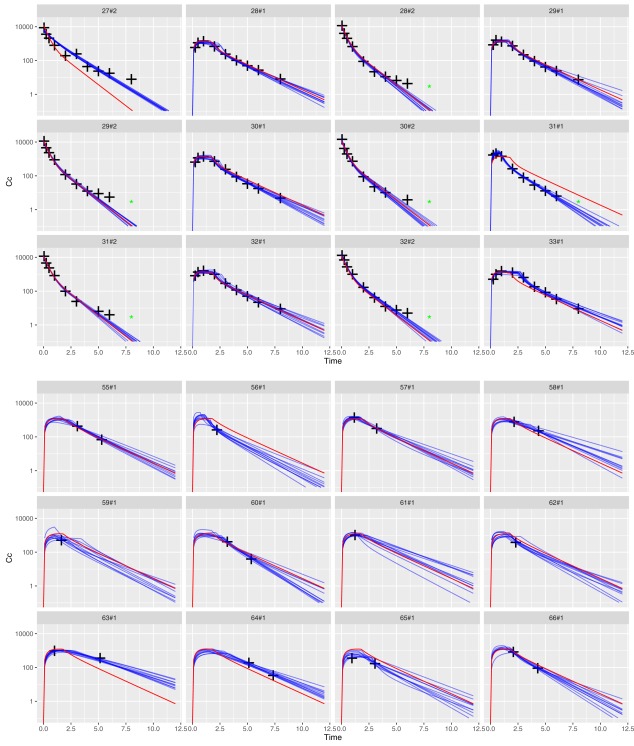
Time‐course of the individual predictions vs. observations using 10 different Markov chains for richly‐sampled (upper panel) and sparsely‐sampled (lower panel) cats. Scatterplot of observed (black crosses) and predicted (continuous lines) robenacoxib levels (log) versus time after dose (hr). Red lines: population predictions. Blue lines: individual predictions derived from each Markov chain. Below limit of quantification levels are represented with green stars. Nomenclature: the first number represents the cat ID; the second number (following #) identifies the dosing route (1 for subcutaneous, 2 for intravenous). The robustness of the model predictions is supported by the good agreement between each of the individual predictions using different Markov chains. IWRES, individual weighted residuals; pdf, probability density function.

**Figure 4:**
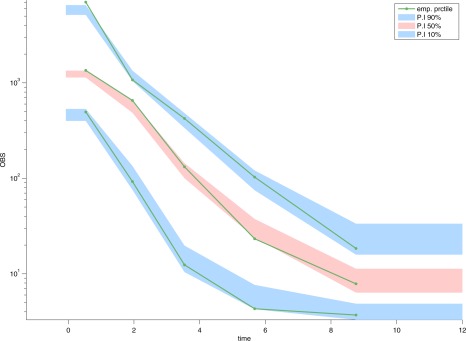
Model validation: Visual Predictive Checks (VPCs). VPCs generated from 500 Monte Carlo simulations. Green lines: 10th, median and 90th percentile of the observed data (OBS). Blue and pink area: 95% confidence interval of the 10th, median and 90th percentile of the prediction interval. F0, fraction absorbed through 0‐order; Ftot, bioavailability; ka, absorption rate; Q, intercompartmental clearance; Tko, absorption duration (0‐order); V1, central compartment volume of distribution; V2, peripheral compartment volume of distribution.

Population PK parameter estimates and their related variances are reported in **Table** 2. The precision of the final model parameters was considered satisfactory (relative standard error <20% for most of the model parameters). The systemic total body CL was estimated to be moderate (0.502 L/kg/h) according to the definition outlined by Toutain and Bousquet‐Mélou.^18^ The global extraction ratio (E) was calculated as E = CL/Q, with cardiac output Q of a cat (mL/kg/min) approximated by the formula: Q=180*
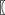
BW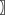 ^−0.195^, where BW is the bodyweight in kg.^18^ The robenacoxib extraction ratio was low (0.058). The s.c. bioavailability was high (78%) with an interindividual variability of 3%. The population steady‐state volume of distribution was estimated at 0.213 L/kg. The absorption constant (K_a_ = 0.68 h^−1^) was lower than the median value for beta (1.10 h^−1^), thereby suggesting that s.c. absorption is the rate limiting step of robenacoxib disposition kinetics in cats. Standard goodness‐of‐fit diagnostic plots are represented in **Figure** 2 (top, middle, and bottom panels) and Supplementary Figure S1. Individual predicted concentration‐time profiles and visual predictive check plots can be found in **Figure** 3 (top and bottom panels) and **Figure** 4 (with Supplementary Figure S2), respectively.

**Table 2:** Point estimates and relative standard error of the mean of PK model parameters with their inter‐individual variability (sequential fitting)

Parameter	Symbol	Unit	Point estimate	Relative standard error (%)	IIV (%)
IV model (initial fitting)					
‐Clearance	CL	L/h/kg	0.50 (§)	§	16
‐Central compartment volume of distribution (ANEST = 0)	V1	L/kg	0.16 (§)	§	41
‐Peripheral compartment volume of distribution	V2	L/kg	0.047 (§)	§	1
‐Inter‐compartmental clearance	Q	L/h/kg	0.065 (§)	§	8
SC model (sequential fitting)					
‐Bioavailability	Ftot	–	0.78 (±0.02)	3	57
‐Fraction absorbed through 0‐order	F0	–	0.50 (±0.05)	10	93
‐Central compartment volume of distribution (ANEST = 1)	V1	L/kg	0.33 (±0.04)	14	41
‐Absorption rate	ka	1/h	0.68 (±0.03)	5	21
‐Absorption duration (0‐order)	Tk0	h	1.78 (±0.1)	5	35

IIV, Inter‐Individual Variability.

§: Not applicable. The fixed‐effects parameters of the disposition function (CL, V1, Q, V2) estimated from the separate fitting of the intravenous dosing were fixed for the following fitting of the subcutaneous and intravenous data. All random effects were estimated from the fitting of the full set of information.

The precision of the final parameter estimates was considered satisfactory (relative standard error <20% for most model parameters).

### Parameter correlations

**Figure 5:**
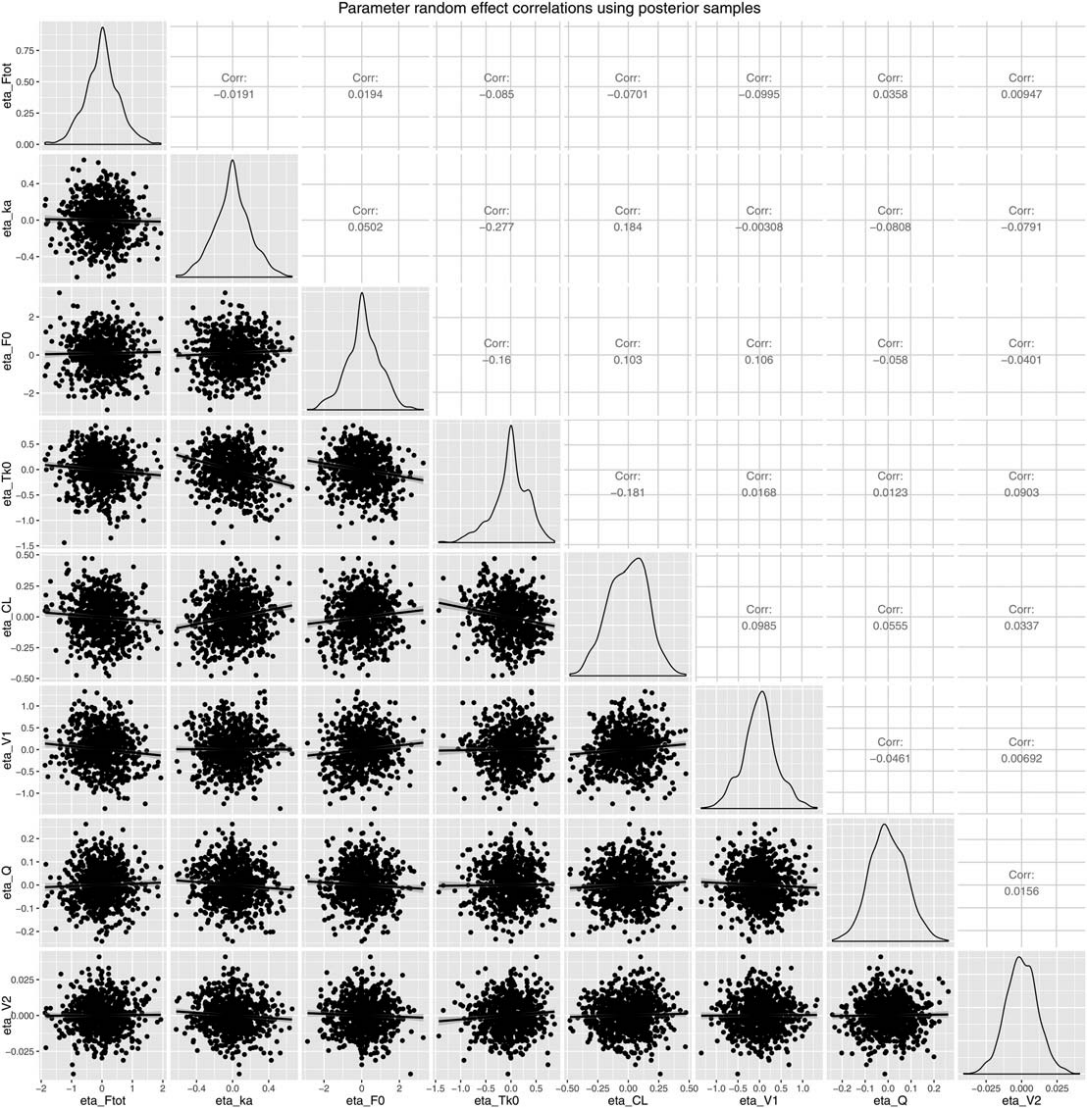
Model parameter correlation matrix using multiple samples from the posterior distribution instead of just the mode (i.e., the EBE).

The correlation matrix of the random effects (i.e., the ηi) is displayed in **Figure** 5. The most notable correlation was found to lie between ka and Tk0 and was, therefore, included in the model structure. No other correlation was found to significantly improve the information criteria. The value of using various samples from the posterior distribution of each individual rather than the mode can readily be observed by comparing the parameter correlation matrix from **Figure** 5 and **Supplementary Figure S5**. Precisely, spurious correlations between model parameters would have been implied looking at the EBEs (e.g., corr(Ftot, V1) = −0.387 or corr(Ftot, CL) = −0.465), whereas these correlations were estimated to be almost flat when using multiple samples from the posterior distribution.

### Effects of demographics and anesthesia on robenacoxib exposure

The log‐transformed estimate of CL as a function of the continuous and categorical covariates is shown in **Supplementary Figure S4** (top and bottom panels, respectively). No significant trend could be detected among age, bodyweight, and CL to explain the between‐subject variability. A modest but not significant difference was observed between the median CL of male and female cats.

After performing the backward elimination, the model with the effect of ANEST on V1 was the model selected. Point estimate for V1 in the clinical cats (ANEST = 1) was 0.33 L/kg compared to 0.16 L/kg in densely sampled laboratory cats.

## DISCUSSION

This modeling work reconciles disposition kinetic information from a large cohort of individuals thereby representing the first population PK study in cats. Previous so‐called population PK studies reported results from a standard two‐stage approach^19^ or derived from a small population (range, 6–8) of intensely sampled cats.^20^, ^21^

In veterinary drug development, dosing regimen selection is commonly based on dose ranging/titration studies that bear a series of limitations: (i) the dose selected as the most effective one is not necessary the optimal dose, as it heavily depends on the power of the design,^22^ and (ii) trials in which the sample size is small frequently lead to the selection of high doses (a phenomenon known as dose dumping), which is relevant to NSAIDs, some of which have low safety indices in cats. As opposed to concentration‐time data, doses *per se* do not contain any pharmacological information. The value of modeling and simulation approaches lies in their ability to use *in silico*/mathematical models for better integration and understanding of quantitative pharmacology. Specifically, mechanism‐based PK/pharmacodynamic modeling techniques provide a quantitative characterization of the relationship between drug exposure in blood and tissues, and the related effect in a biological system. The advantages of using NLME vs. two‐stages approaches (i.e., fixed‐effects modeling approach) include: (i) the analysis of sparse data, which has many applications in veterinary medicine^23^; (ii) the estimation of between‐ and within‐variability in drug exposure and its separation from the measurement error (noise); and (iii) the ability to pool information from diverse sources, which allows for the simultaneous fitting of both i.v. and s.c. routes and the adequate interpretation of PK results. In our specific case with robenacoxib, the simultaneous modeling of the i.v. and s.c. routes unveiled the flip‐flop PKs of the NSAID following extravascular dosing. Two‐stage compartmental modeling of robenacoxib in the cat was carried out for each route separately in four veterinary publications.^6^, ^7^, ^8^, ^9^ The absolute CL determined by our sequential model (0.502 L/kg/h) is in agreement with the previously published CL after i.v. administration CL 0.54 L/kg/h.^7^ However, at that time, the apparent similarity of terminal half‐lives between s.c. and i.v. dosing in Pelligand *et al*.,^7^ albeit observed in a small number of cats, tentatively ruled out a flip‐flop phenomenon. For s.c. administration, the absorption and elimination half‐lives were estimated to be 0.28 hours and 1.87 hours, respectively, in Giraudel *et al*.^10^ Similarly, Pelligand *et al*.^7^ reported half‐lives of 0.28 hours and 1.04 hours for absorption and elimination, respectively. Using the present combined model, the absorption constant (K_a_ = 0.68 h^−1^) was lower than the median value for beta (1.10 h^−1^), thus confirming that a flip‐flop of the absorption and elimination phases occurred with s.c. dosing. These yield absorption and eliminations half‐lives of 0.64 hours and 1.04 hours, respectively. Inversion of the absorption and elimination parameters lead to erroneous estimation of the volume of distributions.^24^ For example, the volume of distribution (Vd) of robenacoxib (s.c.) was estimated to be 0.84 L (95% confidence interval = 0.65–1.03) and 1.130 L (95% confidence interval = 0.949–1.344) in previous publications,^6^, ^7^ which are overestimations of the real volume of distribution of robenacoxib.

This paper reinforces the absolute necessity of investigating the i.v. route of administration during drug development, regardless of the expected route of administration of the final commercial formulation. Not using the i.v. precludes physiological understanding of the disposition kinetics of the investigated drug and later opportunities to use metadata to support a new route or a new dosage regimen.

An important feature of combining data from different sources (preclinical and clinical) lies in the leveraging of information from richly sampled individuals to predict the most likely blood concentration‐time profile in sparsely sampled individuals.^25^, ^26^ The individual EBE estimates (taken as mode of the posterior distribution) of each PK parameters can be used to simulate the most probable individual blood concentration‐time profile for the sparsely sampled cat. These individual profiles then allow evaluation of the time during which concentrations remain above a target pharmacodynamics threshold (for example IC_80_ cyclooxygenase_2_ for efficacy or IC_20_ cyclooxygenase_1_ for safety).^27^ This exposure index can be used as a covariate to support the perioperative clinical efficacy and safety data collected alongside PK samples in the clinical study herein reported.^10^, ^11^ A traditional approach, comparing blood concentrations in clinical cats with *t*‐test, would have been meaningless in the absence of standardized times of sampling. This also demonstrates the interest of meta‐models in drug development, aggregative data from population PK from all stages of development (preclinical studies, field studies, and postmarketing studies) to verify the validity of the population PK model for all populations, especially for the target clinical population.^28^

Through this analysis, we also demonstrated the value of using multiple samples from the posterior distribution instead of just the mode (i.e., the EBEs) to estimate correlations between model parameters. Indeed, the effect of eta‐shrinkage on model parameters estimates was such that spurious correlations could have been suspected for example between Ftot and CL or Ftot and V1 when looking at the scatterplot of correlations using EBEs (**Supplementary Figure S5**). This was confirmed by the fact that the SAEM algorithm could not converge when these correlations were included in the stochastic model, as a likely consequence of structural unidentifiability. Although there is a risk for the EBEs to shrink toward the same population value and the resulting diagnostics to be misleading when data are sparse, our methods that use multiple random samples from the conditional distribution using Markov chains Monte Carlo for assessing correlations and covariate relationship is actually more robust and less sensitive to shrinkage. Indeed, as nicely described by Lavielle and Ribba^16^ the resulting estimator of $\psi_{i}$ is unbiased in the following sense:

$$p\left(\psi_{i}\right)=E\left(p\left(\psi_{i}|y_{i}\right)\right)$$

Therefore, if we were to randomly draw a vector $y_{i}$ of observations for an individual in a population and then generate a vector $\psi_{i}$ using the conditional distribution $\;p\left(\psi_{i}|y_{i}\right)$, the distribution of $\psi_{i}$ would be the population distribution $\;p\left(\psi_{i}\right)$. A direct consequence of this fundamental property is that any diagnostic plot based on these simulated vectors of individual parameters can be used with confidence. Therefore, this case study illustrates the usefulness of using posterior samples rather than the EBEs^29^ to avoid model diagnostic issues related to eta‐shrinkage (shrinkage effect, see refs. 30 and 31).

Differences between experimental and clinical individuals have actually been investigated through the analysis of the ANEST covariate effect on the model parameters (i.e., all anesthetized cats are originated from the population of clinical individuals). In addition, as such, only the effect of ANEST on the volume of distribution of the central compartment proved to be statistically significant as a result of the backward elimination step. The higher central volume of distribution in the cats undergoing general anesthesia could be related to several factors, such as volume expansion due to intraoperative fluid therapy, or the use of anesthesia related‐drugs with vasodilatory properties as acepromazine, propofol, or isoflurane.^4^

The extraction ratio of robenacoxib has been estimated to be rather small in cats (ca. 0.06), such that the hepatic CL can be approximated by the product of the unbound fraction and the intrinsic clearance as follows:

$${CL}_{hepatic}\propto fu\times {Cl}_{int}$$

Because the renal contribution to the systemic CL of robenacoxib is negligible in cats (ca. 15%) (King, J.N., unpublished data) we can also write the following:

$$Cl\propto fu\times {Cl}_{int}$$

"Protein binding in the cat is quite high (> 98%). In these circumstances, and as nicely described by Benet and Hoener,^32^ the free steady‐state concentration, which is pharmacodynamically active, has no dependency on the free fraction. In other words, any change in protein binding due to anesthesia would have little impact on the effect of robenacoxib as a pain relief medication in cats. In addition, the results of our covariate analysis did not suggest any significant change in total clearance in anesthetized cats. We can, therefore, be confident that anesthesia did not substantially affect the intrinsic CL of robenacoxib, unless these changes would be compensated by opposing variations in the unbound fraction of robenacoxib (such that the net effect on total clearance would be null).

No significant effect of gender or age was found on the exposure of robenacoxib in cats. These results can be interpreted with a reasonable degree of confidence because the range of investigated age (0.34–6.2 years old) and bodyweight (1.8–5.7 kg) was sufficiently wide. A correlate of this may be that there is no need for dosing adjustment based on population demographics (age and bodyweight). This conclusion is different from the situation in dogs, in which bodyweight had a significant effect on apparent robenacoxib CL and volume of distribution.^1^ Anesthesia itself does not seem to have a significant influence on robenacoxib exposure in blood either, which also speaks for no dose adjustment in the context of anesthesia.

The Population Fisher Information Matrix could help identify the “best” vector of sampling points from an estimation/uncertainty viewpoint. However, this sampling scheme might be impossible to implement in the practice of veterinary anesthesia because the timing of the sampling for the sparsely sampled cats was dictated by the time of the end of the anesthesia and 2 hours thereafter and the time of drug administration (which varied randomly between cats and groups). The individual predictions of the plasma concentration‐time curves for the sparsely sampled cats will support efficacy and safety data in a follow‐up publication highlighting the risk/benefits characterization of preoperative vs. postoperative NSAIDs administration, including the case of robenacoxib.^10^, ^11^

The outcome of the covariate analysis should be interpreted with caution for several reasons. First, this experimental design is vulnerable to a possible confounding effect. Indeed, all sparse data came from anesthesia/perioperative conditions. For age, the range of values from the perioperative study overlaps with that of other studies, but for gender, only female cats have been included in the perioperative study and have undergone sparse sampling around anesthesia. As such, the effect of anesthesia on robenacoxib disposition kinetics in male cats cannot be discussed and, therefore, the estimates of exposure from sparse animals cannot be directly compared with intensively sampled cats. Second, the retrospective nature of the experimental design did not allow for balancing the dataset with regard to the allocation ratio of sparsely sampled cats to more intensely sampled cats. This may have resulted in a study underpowered to detect differences between groups. Last, our interpretation of the covariate analysis relates to the systemic PK so no conclusions can be drawn on the influence of age, gender, bodyweight, and anesthesia at the target site of action (most likely the central nervous system in the context of perioperative pain management).

## Supporting information

Supporting Information

Supporting Information

Supporting Information

Supporting Information

Supporting Information

Supporting Information

Supporting Information

Supporting Information
