# Supplementary figures and images for: Modeling of Large Pharmacokinetic Data Using Nonlinear Mixed‐Effects: A Paradigm Shift in Veterinary Pharmacology. A Case Study With Robenacoxib in Cats
Source: CPT Pharmacometrics Syst Pharmacol. 2016 Oct 22;5(11):625–35. doi: 10.1002/psp4.12141 (PMC5193001; doi:10.1002/psp4.12141)

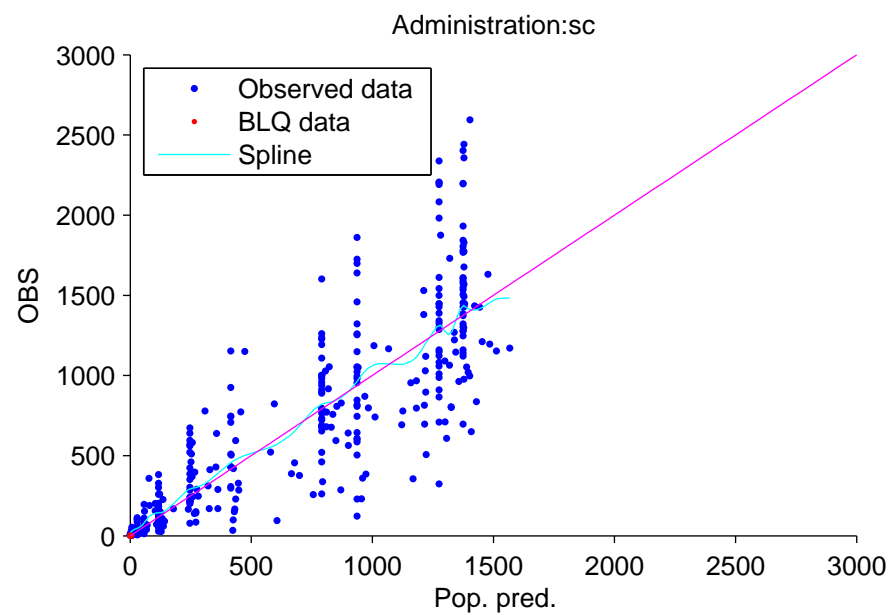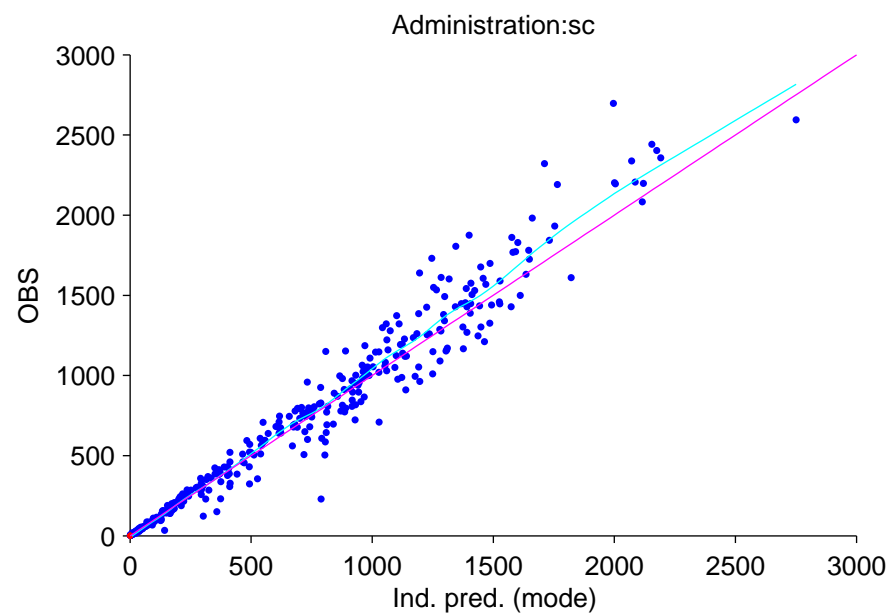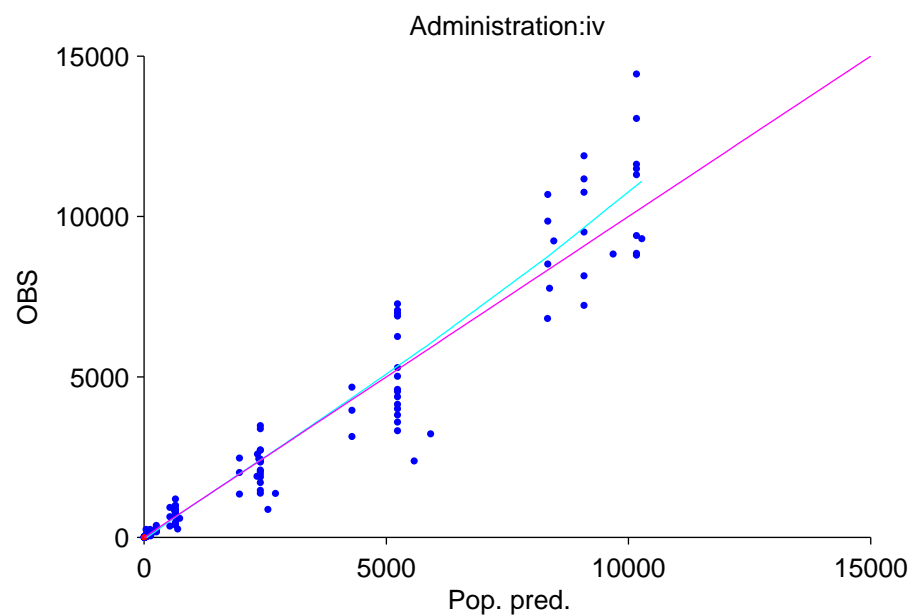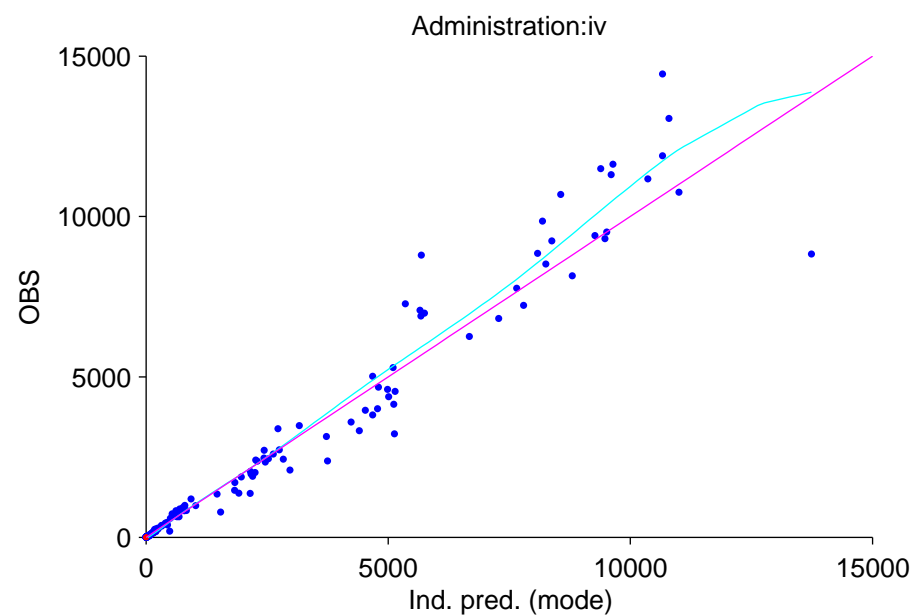

Supplement: Supplementary file 2 — Supporting Information [file PSP4-5-625-s002.pdf]

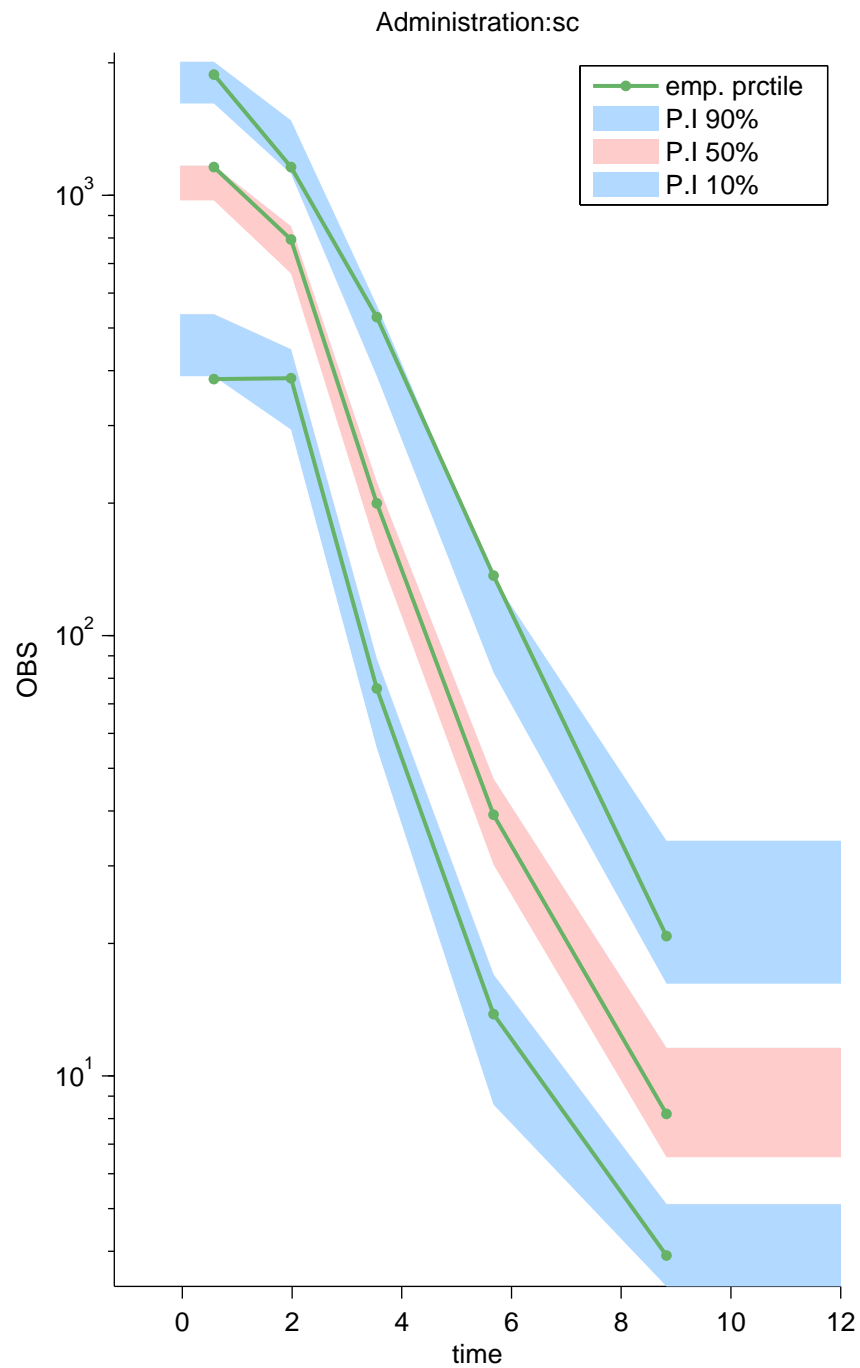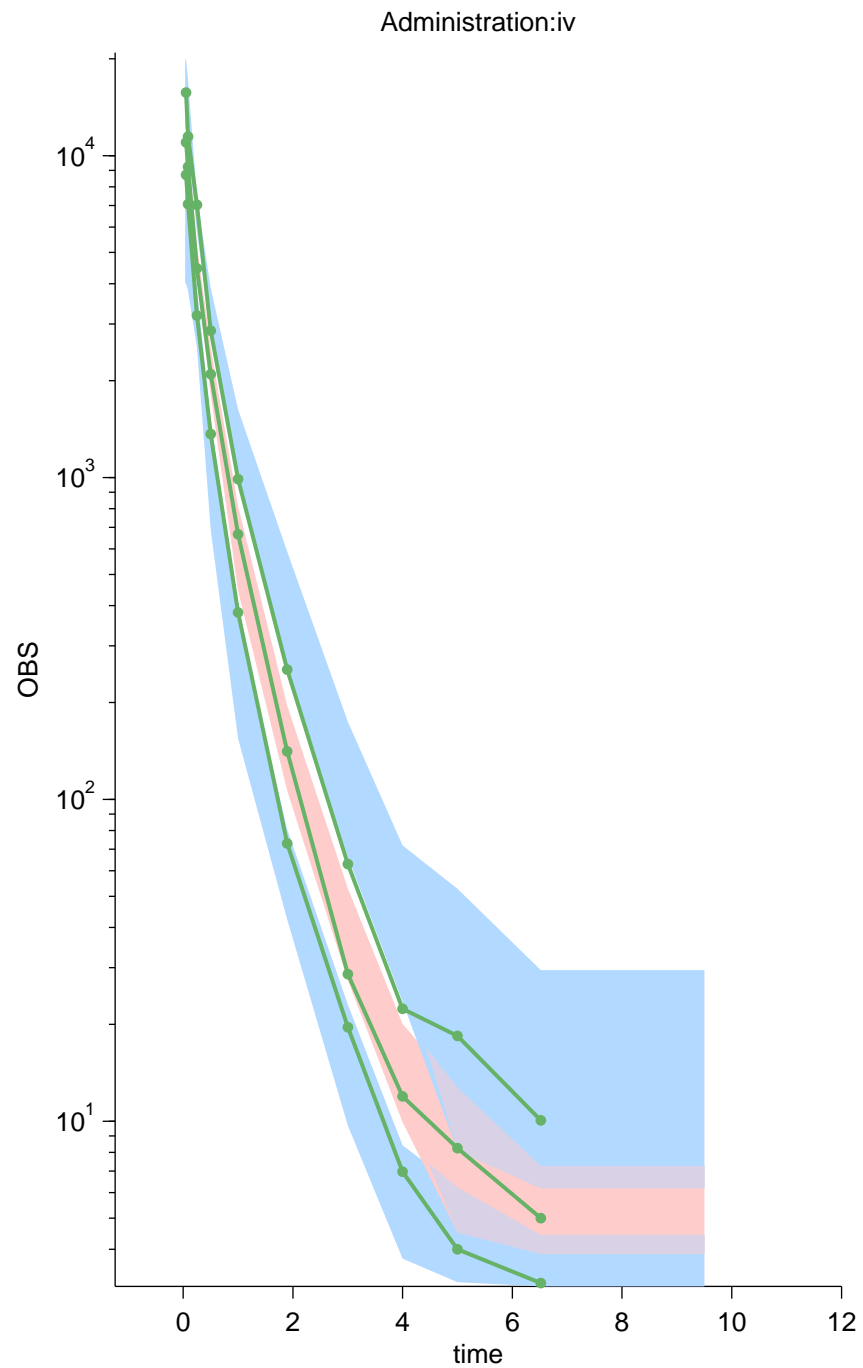

Supplement: Supplementary file 3 — Supporting Information [file PSP4-5-625-s003.pdf]

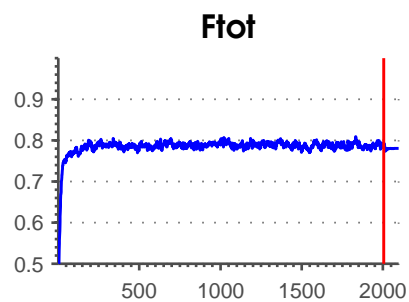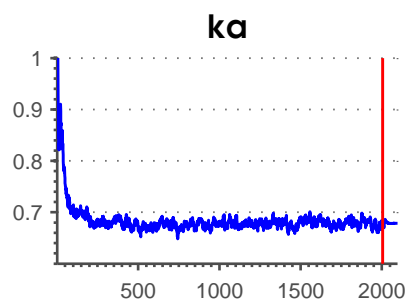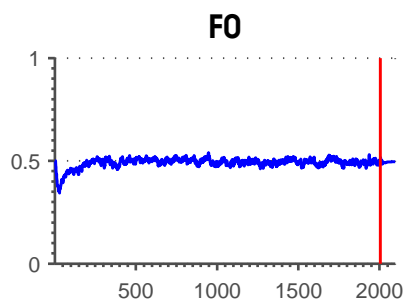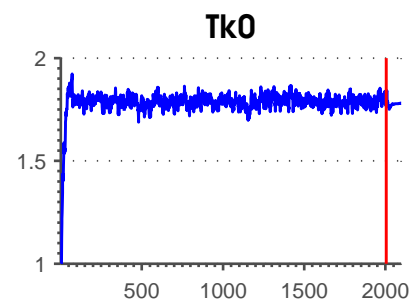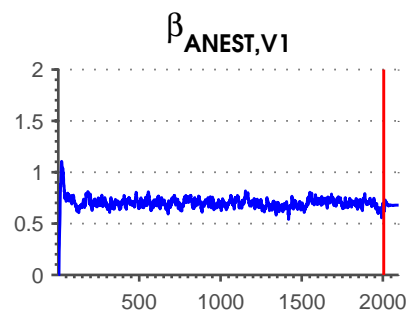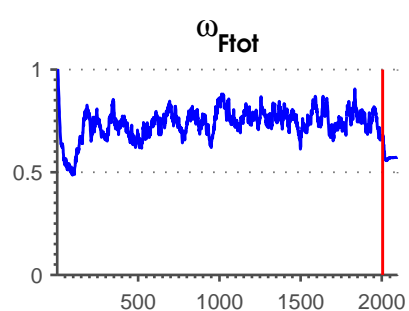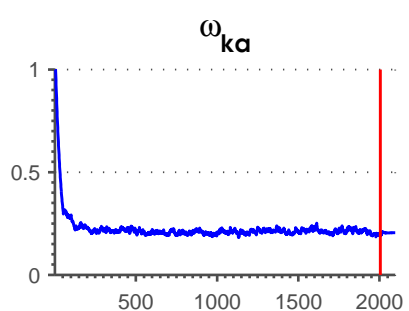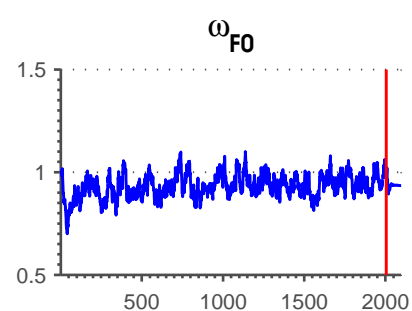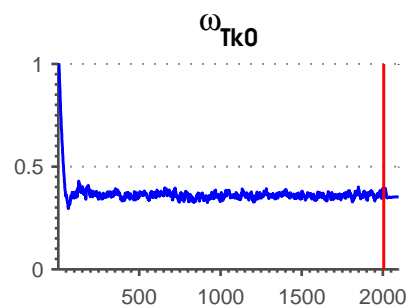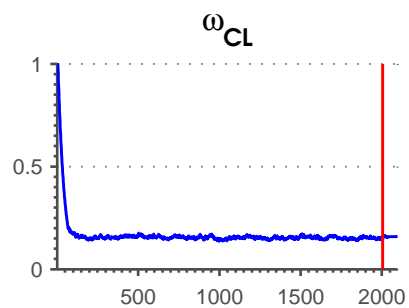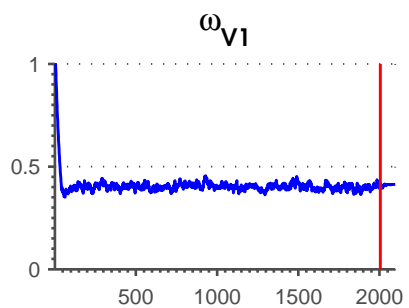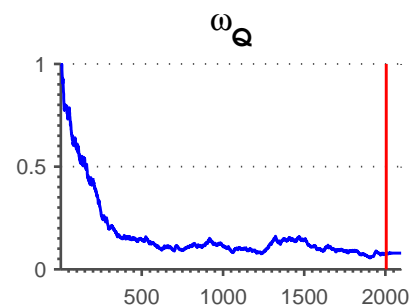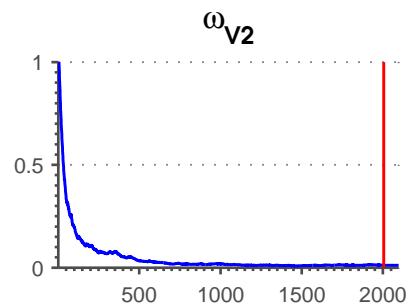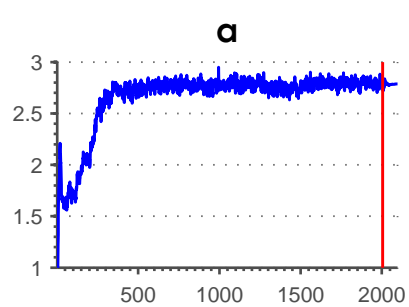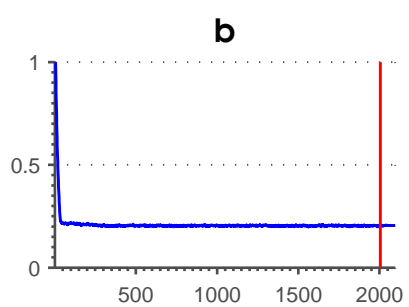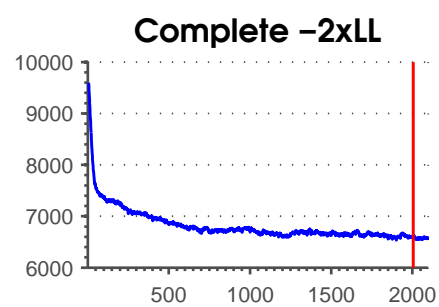

Supplement: Supplementary file 4 — Supporting Information [file PSP4-5-625-s004.pdf]
