# Supplementary material for: Modeling of Large Pharmacokinetic Data Using Nonlinear Mixed‐Effects: A Paradigm Shift in Veterinary Pharmacology. A Case Study With Robenacoxib in Cats
Source: CPT Pharmacometrics Syst Pharmacol. 2016 Oct 22;5(11):625–35. doi: 10.1002/psp4.12141 (PMC5193001; doi:10.1002/psp4.12141)
Supplement: Supplementary file 6 — Supporting Information [file PSP4-5-625-s006.pdf]

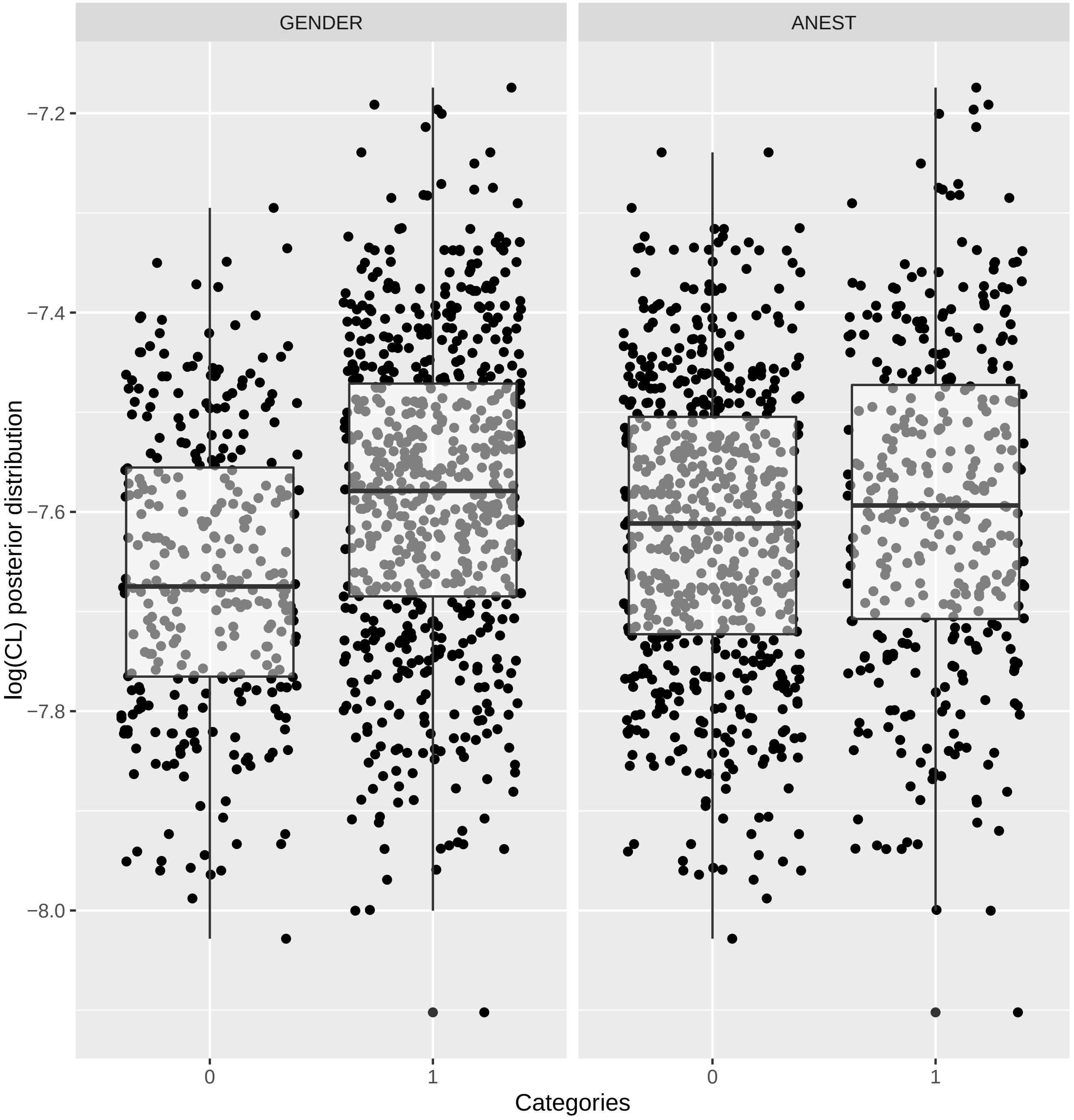

Script: ~/Projects/Clusters/Jonathan/Robenacoxib/Nov2015/main_robenacoxib2.R

Output: Log.transformed.CL.as.function.of.categorical.covariates

Execution:

08−Aug−2016−21:23:42
